# Extracellular HSP90 Machineries Build Tumor Microenvironment and Boost Cancer Progression

**DOI:** 10.3389/fcell.2021.735529

**Published:** 2021-10-14

**Authors:** Pietro Poggio, Matteo Sorge, Laura Seclì, Mara Brancaccio

**Affiliations:** Department of Molecular Biotechnology and Health Sciences, University of Torino, Torino, Italy

**Keywords:** eHSP90 (Extracellular Heat Shock Protein 90), tumor microenvironment, CDC37, Clusterin, CHORDC1, Morgana, LRP1, TLR (toll like receptors)

## Abstract

HSP90 is released by cancer cells in the tumor microenvironment where it associates with different co-chaperones generating complexes with specific functions, ranging from folding and activation of extracellular clients to the stimulation of cell surface receptors. Emerging data indicate that these functions are essential for tumor growth and progression. The understanding of the exact composition of extracellular HSP90 complexes and the molecular mechanisms at the basis of their functions in the tumor microenvironment may represent the first step to design innovative diagnostic tools and new effective therapies. Here we review the impact of extracellular HSP90 complexes on cancer cell signaling and behavior.

## Introduction

In order to survive and proliferate in a highly stressful environment cancer cells upregulate chaperone expression and actively release chaperones in the extracellular milieu to guarantee their survival and sustain their aberrant evolution toward malignancy (Velichko et al., 2013).

The Heat Shock Protein 90 (HSP90) is a highly conserved and ubiquitously expressed chaperone essential for cell survival. It represents the 1–2% of the total protein content and plays multiple roles, ranging from protein folding to buffering protein denaturation and assisting protein conformational changes (Schopf et al., 2017). Overexpression of HSP90 promotes cell survival, sustains oncogenic signal transduction, cell proliferation and migration (Jaeger and Whitesell, 2019). High HSP90 levels are frequent in human cancer and correlates with poor prognosis (Cheng et al., 2012), with the exception of some specific tumor contexts (Nanbu et al., 1998).

HSP90 comes in two isoforms, the stress-induced HSP90α and the constitutively expressed HSP90β (Sreedhar et al., 2004). In physiological conditions, normal cells express more HSP90β than HSP90α (Ullrich et al., 1989) while in cancer cells HSP90α doubles HSP90β expression level (Zuehlke et al., 2015; Dong et al., 2016). HSP90α underexpression correlates with favorable outcome in some cancer types (Gallegos Ruiz et al., 2008; Buffart et al., 2012).

## The Intracellular HSP90 Machinery

**FIGURE 1:**
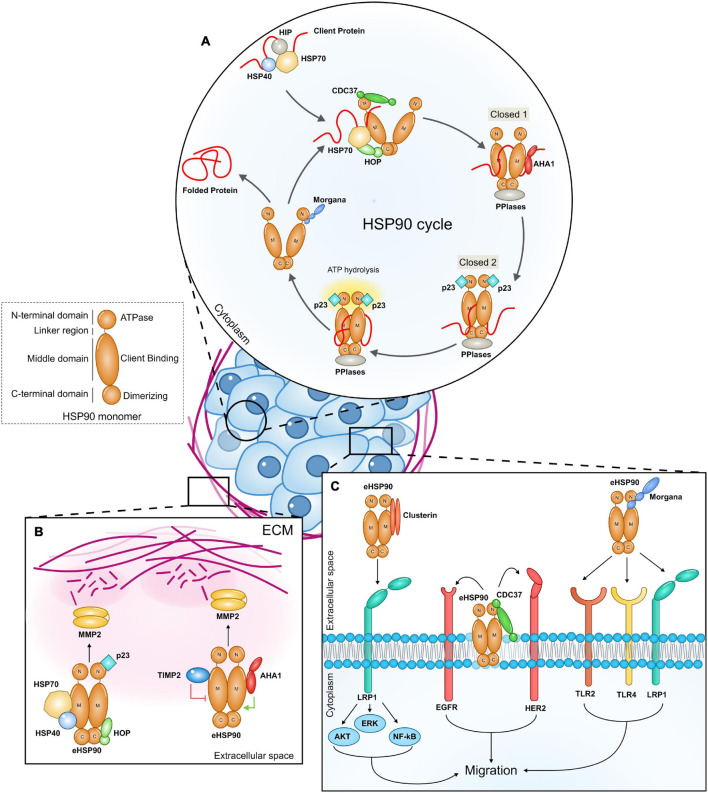
**(A)** The intracellular HSP90 cycle. The figure highlights the role of co-chaperones that participate to the HSP90 intracellular machinery and that have been also found to be part of extracellular HSP90 complexes. The client protein is initially bound by the HSP70/HSP40 complex and then stabilized by HSP70-interacting protein (HIP). The client protein is loaded onto HSP90 from the HSP70/HSP40 protein complex thanks to the adaptor protein HOP (HSP90–HSP70 organizing protein). The co-chaperone CDC37 (cell-division-cycle 37 homolog) is required by HSP90 to bind client kinases. As the client protein is loaded, other co-chaperones, peptidyl-prolyl-isomerase and PPIases (FKBP51, FKBP52) join the complex while the HSP70, HIP, and HOP are released. AHA1 (activator of HSP90 ATPase homolog 1) binding induces HSP90 to switch from the open state to the closed 1 state, while p23 induces ATP hydrolysis and the transition toward the closed 2 conformation. The co-chaperone Morgana interacts with HSP90 in the ADP-bound state. **(B,C)** Extracellular HSP90 (eHSP90) complexes promote cancer cell migration. eHSP90 released by cancer cells binds to different secreted co-chaperones in the extracellular milieu. eHSP90 complexes interact with extracellular client proteins favoring ECM remodeling **(B)** and with surface receptors **(C)**, triggering intracellular signal transduction (ECM, extracellular matrix; M, HSP90 Middle domain; C, HSP90 C terminal domain; N, HSP90 N terminal domain).

HSP90 forms a flexible homodimer and binds to its clients, promoting modifications in their structure in an ATP dependent manner. A single monomer of HSP90 is composed by three domains: an N-terminal ATP-binding domain, a middle domain and a C-terminal dimerization domain. In order to fold client proteins, the HSP90 dimer undergoes conformational rearrangements switching between a closed N-terminal conformation and an open one (Figure 1A). Co-chaperones bind sequentially and reversibly to HSP90 to regulate its conformational changes, its ATPase activity and to confer specificity to clients. Client proteins are bound by the HSP70/HSP40 complex, which is stabilized by HSP70-interacting protein (HIP). The HSP90/HSP70 organizing protein (HOP) facilitates the interaction between HSP90 and HSP70 and the translocation of the client protein to HSP90 (Schopf et al., 2017). The HSP70/HOP complex stabilizes HSP90 in the open conformation and inhibits its ATPase activity. This state primes the binding of peptidyl-prolyl isomerases (PPIases), such as FK506 Binding Proteins (FKBP51 and FKBP52), which support cycle progression. Activator of HSP90 ATPase homolog 1 (AHA1) weakens the interaction between HOP and HSP90, promoting HOP and HSP70 release and the transition toward the N-terminal closed state (closed 1). The binding of p23 displaces AHA1, inducing the switch to a completely closed state (closed 2), which is followed by ATP hydrolysis, the return to the open conformation and the release of the client together with p23 and PPIases (Rohl et al., 2013). Cell division cycle 37 homolog (CDC37) is a crucial HSP90 ATPase-inhibiting co-chaperone which stabilizes the HSP90 open form and recruits kinase clients (Siligardi et al., 2002; Taipale et al., 2012). The protein phosphatase and co-chaperone PP5 associates with HSP90 N-terminal domain and dephosphorylates HSP90 and CDC37, regulating client protein processing (Li et al., 2012). The ability to interact with HSP90 N-terminal domain is also a property of CHORD (Cysteine and Histidine Rich Domain) containing proteins. Indeed, Morgana and Melusin bind to HSP90 preferentially when it is in an ADP-bound state (Gano and Simon, 2010), favoring the protection of cells from different stress stimuli (Michowski et al., 2010; Ferretti et al., 2011; Sorge and Brancaccio, 2016).

## Extracellular HSP90

HSP90 emerged as one of the more abundant secreted chaperones in normal cells as keratinocytes, dermal fibroblasts and neurons (Sidera et al., 2004; Sidera and Patsavoudi, 2008; Li et al., 2012, 2013; Bhatia et al., 2016). The lack of a secretory signal peptide and experimental data (Santos et al., 2017; Kim et al., 2018) indicate that chaperone secretion relies on unconventional mechanisms. HSP90 secretion can be induced by various environmental stimuli. Hypoxia-inducible factor 1 alpha (HIF-1α) stabilization in hypoxic conditions (Li et al., 2007; Sahu et al., 2012) and extracellular signal-regulated kinase (ERK) pathway activation in response to oxidative stress (Liao et al., 2000) are both able to stimulate HSP90 secretion. In the extracellular compartment, HSP90 may exist in a soluble form, but it can be also found bound to phospholipids on plasma membrane and even on the surface or inside exosomes (Shevtsov et al., 2020). eHSP90α promotes cell migration and invasion, crucial processes in tissue morphogenesis, embryonic development, and wound healing processes (Wong and Jay, 2016).

## eHSP90 in Cancer Progression

The release of HSP90, mainly HSP90α, from cancer cells and its role in promoting cancer cell survival, migration, invasion, and stemness through autocrine mechanisms is well established (Liu et al., 2019; Secli et al., 2021). Cells of the tumor microenvironment also express HSP90 receptors and several studies reported the binding of HSP90 to their surface (Calderwood et al., 2016). eHSP90α may mediate fibroblast migration and conversion to cancer associated fibroblasts (Bohonowych et al., 2014; Tang et al., 2019) as well as migration and tubulogenesis in lymphatic endothelial cells (Hou et al., 2021). By interacting with immune cells in the tumor microenvironment, HSP90 may also exert anti-tumoral functions. Indeed, HSP90 is released from cancer cells bound to oncopeptides and facilitates their presentation to antigen presenting cells, stimulating anti-cancer immunity and tumor regression (Calderwood et al., 2016). Nevertheless, the amount of HSP90α in patient serum correlates with tumor progression and the presence of distant metastasis (Liu et al., 2019, 2021).

eHSP90 pro-tumoral activity occurs with different mechanisms. It can activate extracellular clients, promoting the formation of a favorable milieu for cell migration or it can bind to surface receptors stimulating cell survival, movement and aggressiveness (Li et al., 2013).

eHSP90 clients include several proteins involved in extracellular matrix remodeling, like metalloproteinases (MMP2 and MMP9) (Song et al., 2010; Stellas et al., 2010; Sims et al., 2011; Baker-Williams et al., 2019), the pro-form of tissue plasminogen activator (tPA) (McCready et al., 2010), the lysil oxidase-like protein 2 (LOXL2), and fibronectin (Boel et al., 2018; Chakraborty et al., 2020). This suggests that eHSP90 has the potential to regulate matrix deposition and stiffness, thus influencing cancer aggressiveness (De Maio and Vazquez, 2013; Kai et al., 2016; Wong and Jay, 2016).

eHSP90 binds to several cell surface receptors and triggers signal transduction pathways that contribute to malignancy. It interacts with Toll-like receptor 4 (TLR4) and signals via Proto-oncogene tyrosine-protein kinase (SRC) to focal adhesion kinase (FAK), which is critical for cell motility. eHSP90-mediated TLR4 activation may also induce the transactivation of the epithelial growth factor receptor (EGFR), further contributing to malignancy (Thuringer et al., 2011). Binding to the extracellular domain of HER2, eHSP90 unleashes SRC, ERK, and RAC-alpha serine/threonine-protein kinase (AKT) pathways (Belsches-Jablonski et al., 2001; Sidera et al., 2008). eHSP90 also interacts with low-density lipoprotein receptor-related protein 1 (LRP1). LRP1 is a ubiquitous endocytic receptor that recognizes several ligands and transduces signals, regulating tissue remodeling, cell survival and inflammatory reactions (Calderwood, 2018). eHSP90 binding to LRP1 activates ERK, AKT, and NF-κB pathways and induces ephrin type-A receptor 2 (EPHA2) recruitment and activation, promoting lamellipodia formation and cancer cell motility and invasion (Chen et al., 2010; Gopal et al., 2011; Tsen et al., 2013; Nagaraju et al., 2015).

Interestingly, a small eHSP90α-specific fragment, located at the boundary between the linker and the M domain, is sufficient to reproduce the ability of the entire chaperone to induce cell migration trough LRP1 (Cheng et al., 2011; Zou et al., 2017). Nevertheless, increasing evidence indicates that eHSP90 pro-tumorigenic activity is regulated and directed by secreted co-chaperones (Table 1). This suggests that co-chaperones may regulate the accessibility of the HSP90 pro-motility fragment, besides conferring specificity to clients and receptors.

**TABLE 1:** Roles of extracellular HSP90 co-chaperones.

**Co-chaperone**	**Intracellular functions**	**Role of eHSP90 complexes in cancer progression**	**References**
CDC37	Stabilization of HSP90 open conformation; presentation of kinases clients to HSP90	Induction of cancer cell migration through HER2 and EGF receptors	El Hamidieh et al., 2012; Baker-Williams et al., 2019
AHA1	Induction of HSP90 ATPase activity	Competition with TIMP2 for the activation of the HSP90-MMP2 complex	Baker-Williams et al., 2019
p23	Stabilization of HSP90 closed 2 state	MMP2 activation	Sims et al., 2011
HOP	Stabilization of HSP90 open conformation; transfer of client proteins from HSP70 to HSP90	MMP2 activation	Sims et al., 2011; Hajj et al., 2013; Baker-Williams et al., 2019
HIP	Transfer of client proteins from HSP70 to HSP90	Not investigated	Crescitelli et al., 2020
HSP40	Stimulation of the association between HSP70 and HIP; transfer of client proteins from HSP70 to HSP90	MMP2 activation	Gehrmann et al., 2005; Sims et al., 2011
HSP70	ATP-dependent molecular chaperone; binding and transfer of client proteins to HSP90	MMP2 activation; TLR4 Activation	Lee et al., 2006; Sims et al., 2011; Guzhova et al., 2013; De Maio, 2014
PP5	HSP90 phosphatase	Not investigated	Baker-Williams et al., 2019
FKBP51/FKBP52	Peptidyl-prolyl-isomerase involved in client protein maturation	Not investigated	Criado-Marrero et al., 2018
Morgana	Form a complex with ADP-bound HSP90; regulation of intracellular signaling pathways	Induction of cancer cell migration through TLR4, TLR2, and LRP1 receptors	Seclì et al., 2021
Clusterin	Mediation of intracellular proteostasis	Induction of cancer cell migration through LRP1 receptor	Tian et al., 2019

## eHSP90 Complexes Remodel Tumor Microenvironment

Four co-chaperones, namely HSP70, HSP40, HOP, and p23 were found in complex with eHSP90α in breast cancer cell conditioned medium. These factors increase eHSP90α binding to MMP2, enhancing MMP2 activation and resulting in cancer cell invasion (Figure 1B). HSP70 depletion from conditioned media or its pharmacological inhibition impairs eHSP90-mediated MMP2 activation (Sims et al., 2011). Similar multichaperone complexes have been found also in other contexts. eHSP90α and eHSP70 released on the surface of extracellular vesicles are responsible for tumor-induced muscle wasting. Both HSP70 and HSP90α are required to activate TLR4 on muscle cells to induce cachexia (Zhang et al., 2017), however it is not clear if a direct interaction occurs. Experiments in neuroblasts demonstrate that eHOP, eHSP70, and eHSP90 cooperate to influence cell migration, suggesting the possibility that these chaperones generate a functional complex (Miyakoshi et al., 2017). In these studies, the presence of further components has not been investigated.

In fibrosarcoma cell conditioned medium, eHSP90α requires an articulated competing system between co-chaperones to induce client maturation (Baker-Williams et al., 2019). Researchers identified the Tissue inhibitor of metalloproteinase 2 (TIMP2), an endogenous inhibitor of MMPs, as a new extracellular co-chaperone of eHSP90α. TIMP2 forms a complex with eHSP90α, PP5 and HOP and inhibits eHSP90α ATPase activity by binding its middle domain. In this context, TIMP2 functions as a scaffolding co-chaperone that loads MMP2 to eHSP90α, keeping MMP2 in a transiently inhibited state. AHA1 competes with TIMP2 for the binding to the HSP90α-MMP2 complex (Figure 1B). Indeed, TIMP2, and AHA1 occupy the same epitope on the M-domain of eHSP90α and AHA1 may displace TIMP2 from the HSP90α-MMP2 complex, promoting the full activation of the metalloproteinase and inducing matrix degradation. The evidence for sequential events influencing eHSP90 complex composition and client activation suggests the existence of bona fide eHSP90 machineries. It remains a matter of debate if HSP90 extracellular functions are dependent on its ATPase activity. Many studies have proposed and demonstrated that eHSP90 can chaperone clients in an ATP independent manner (Cheng et al., 2011; Sims et al., 2011; McCready et al., 2014), however Baker-Williams et al. (2019) proved that eHSP90 needs ATP hydrolysis to properly fold MMP2. Since cells can actively secrete ATP in response to a variety of stressful stimuli (Di Virgilio, 2021), it is possible that, at least in some contexts, eHSP90 binds, and hydrolases ATP to carry out its extracellular duties.

## eHSP90 Complexes Activate Cancer Cell Surface Receptors

Several co-chaperones assist and regulate eHSP90 binding to cell surface receptors, likely conferring binding specificity and/or promoting specific signaling events.

CDC37 has been found secreted by triple negative breast cancer cells and localized on the cell surface (El Hamidieh et al., 2012). Similarly to its intracellular counterpart, eCDC37 acts as a co-factor of eHSP90. The HSP90 isoform involved in this specific complex has not been investigated. The eHSP90/eCDC37 complex interacts on the cell surface with HER2 and EGF receptors allowing breast cancer cell migration (Figure 1C). Indeed, treatment with anti-CDC37 antibodies impairs cancer cell migration and *in vivo* administration of an HSP90 blocking antibody, able to disrupt the HSP90-CDC37 complex, inhibits metastasis formation (Stellas et al., 2007, 2010; El Hamidieh et al., 2012).

Clusterin is a secreted glycoprotein that functions as an ATP-independent extracellular chaperone for several clients in extracellular fluids, acting by inhibiting amorphous protein aggregation and contributing to the clearance of unfolded proteins (Jenne and Tschopp, 1989; Calero et al., 1999; Wyatt et al., 2011). LDL Receptor Related Protein 2 (LRP2) and Plexin A4 have been identified as Clusterin receptors in brain (Kang et al., 2014, 2016). It has been demonstrated that Clusterin interacts with eHSP90α and increases its ability to associate with LRP1. The Clusterin/HSP90α complex binds to LRP1 with higher affinity in respect to eHP90α alone and potentiates the signal, increasing breast cancer cell migration (Figure 1C). Clusterin and eHSP90α synergistic signaling induces E-cadherin downregulation and increases the expression of N-cadherin, Snail, Slug, and Zeb1, promoting epithelial to mesenchymal transition. *In vivo* co-administration of eHSP90α and Clusterin in mice carrying breast cancer cells derived tumors significantly increases metastasis (Tian et al., 2019).

Morgana is a HSP90 co-chaperone coded by the CHORDC1 gene. Inside the cells, Morgana regulates signal transduction by binding and inhibiting Rho kinases I and II (Ferretti et al., 2010; Fusella et al., 2014) and promotes NF-kB activation (Fusella et al., 2017). It is also involved in microtubule polymerization (Palumbo et al., 2020), EGF receptor trafficking (Haag et al., 2020), and extracellular vesicle secretion (Urabe et al., 2020). We recently found that Morgana is secreted by several cancer cells through an unconventional pathway and it associates with HSP90 in the extracellular milieu. The involvement of a specific HSP90 isoform has not been yet clarified. Cancer cells downregulated for Morgana migrate less than control cells and the addition of a recombinant Morgana in the medium totally rescues the defect in cell migration. HSP90 blocking antibodies revert this ability, suggesting that the eMorgana works in concert with HSP90 to promote cell migration. We found that TLR4, TLR2, and LRP1 are all required for eMorgana function (Figure 1C). Indeed, the impairment of the activity of these receptors by blocking antibodies or RNA interference makes the cells insensitive to Morgana pro-migratory signals. The available data indicate that Morgana binds directly to TLR2, while it requires additional components, present in the cancer cell conditioned medium, to bind to LRP1. The interaction between Morgana and TLR4 remains elusive and the possibility that TLR4 may participate to Morgana signal transduction thank to a cross-talk with TLR2 and LRP1 has to be taken into account. The fact that cancer cell migration in presence of Morgana blocking antibodies is not further repressed by inhibiting HSP90 suggests that the extracellular complex containing HSP90 and Morgana is the main responsible for migration, at least in a subgroup of cancer cells (Seclì et al., 2021).

## eHSP90 Complexes as Therapeutic Targets

Eighteen HSP90 inhibitors have entered clinical trials but none has been approved by the FDA, mainly due to associated toxicity (Xiao and Liu, 2020). Several pre-clinical trials are now exploring the possibility to selectively inhibit the extracellular HSP90 (Tsutsumi et al., 2008; Wang et al., 2009; Song et al., 2010). However, since eHSP90 may also induce anti-cancer immunity (Calderwood et al., 2016), the opportunity to specifically target eHSP90 pro-tumorigenic complexes would further improve the therapeutic value of these approaches. This strategy is achievable by targeting co-chaperones present in pro-tumorigenic complexes (Barrott and Haystead, 2013; Edkins et al., 2018; D’Annessa et al., 2020). *In vitro* and *in vivo* experiments have proved that targeting extracellular co-chaperones have the potential to inhibit cancer progression. Treatments with antibodies against AHA1 or TIMP2 are able to inhibit MMP2 activity (Baker-Williams et al., 2019). The use of cell impermeable anti-CDC37 antibody compromises the invasiveness of breast cancer tumor cells (El Hamidieh et al., 2012). Similar effects have been described *in vitro* and in cancer preclinical models for the monoclonal antibody against eHSP90 (4C5), able to disrupt the eCDC37/eHSP90/HER2 or EGFR complex (Stellas et al., 2007, 2010; El Hamidieh et al., 2012). A monoclonal antibody against Morgana (mAb 5B11B3) has been recently identified as an inhibitor of cancer cell migration both *in vitro* and in pre-clinical models. mAb 5B11B3 systemic treatment in immunocompromised tumor-bearing mice inhibits cancer cell intravasation and metastasis. In syngeneic cancer mouse models, in addition to reducing metastases, mAb 5B11B3 abates tumor growth by promoting anti-cancer immunity mediated by macrophages and CD8^+^ T lymphocytes (Seclì et al., 2021).

## Discussion

While the role of eHSP90 as a promoter of cancer progression is well established, the involvement of its extracellular co-chaperones remains poorly investigated. Available data suggest that eHSP90 binds to extracellular co-chaperones, forms different complexes and generates machineries with specific missions in the outside. Little is known on the dynamic of eHSP90 complex formation and on their possible cooperation in activating cell surface receptors and extracellular clients. It has been demonstrated that inside cancer cells, HSP90 can form stable aberrant multi-chaperone complexes that facilitate cell survival (Rodina et al., 2016). In the extracellular medium, similar complexes could work as platforms, clustering surface receptors and regulating their transactivation, or acting as scaffolds for extracellular matrix remodeling. A deep understanding of the eHSP90 interaction network appears a promising starting point to develop new diagnostics tools and provide potential targets for drug intervention in cancer.

## Author Contributions

All authors wrote the manuscript and approved the contents for publication.

## Conflict of Interest

The authors declare that the research was conducted in the absence of any commercial or financial relationships that could be construed as a potential conflict of interest.

## Publisher’s Note

All claims expressed in this article are solely those of the authors and do not necessarily represent those of their affiliated organizations, or those of the publisher, the editors and the reviewers. Any product that may be evaluated in this article, or claim that may be made by its manufacturer, is not guaranteed or endorsed by the publisher.
